# Biosensing Based on Nanoparticles for Food Allergens Detection

**DOI:** 10.3390/s18041087

**Published:** 2018-04-04

**Authors:** Lidia Nazaret Gómez-Arribas, Elena Benito-Peña, María del Carmen Hurtado-Sánchez, María Cruz Moreno-Bondi

**Affiliations:** Departamento de Química Analítica, Facultad de Ciencias Químicas, Universidad Complutense de Madrid, 28040 Madrid, Spain; lidianazaretgomez@ucm.es (L.N.G.-A.); elenabp@ucm.es (E.B.-P.); mahurt03@ucm.es (M.d.C.H.-S.)

**Keywords:** biosensor, biosensing, allergen, nanotechnology, gold nanoparticles, carbon nanotubes, quantum dots, food

## Abstract

Food allergy is one of the major health threats for sensitized individuals all over the world and, over the years, the food industry has made significant efforts and investments to offer safe foods for allergic consumers. The analysis of the concentration of food allergen residues in processing equipment, in raw materials or in the final product, provides analytical information that can be used for risk assessment as well as to ensure that food-allergic consumers get accurate and useful information to make their food choices and purchasing decisions. The development of biosensors based on nanomaterials for applications in food analysis is a challenging area of growing interest in the last years. Research in this field requires the combined efforts of experts in very different areas including food chemistry, biotechnology or materials science. However, the outcome of such collaboration can be of significant impact on the food industry as well as for consumer’s safety. These nanobiosensing devices allow the rapid, selective, sensitive, cost-effective and, in some cases, in-field, online and real-time detection of a wide range of compounds, even in complex matrices. Moreover, they can also enable the design of novel allergen detection strategies. Herein we review the main advances in the use of nanoparticles for the development of biosensors and bioassays for allergen detection, in food samples, over the past few years. Research in this area is still in its infancy in comparison, for instance, to the application of nanobiosensors for clinical analysis. However, it will be of interest for the development of new technologies that reduce the gap between laboratory research and industrial applications.

## 1. Introduction

Food allergy is defined as an immunological reaction resulting from consumption or another contact with a given food [1]. Food allergens are specific components of food or ingredients within the food, usually proteins but sometimes chemical haptens, which are recognized by specific immune cells triggering the immune reaction and resulting in characteristic symptoms ranging from mild to life-threatening [2,3].

In clinical terms, food hypersensitivity can be classified into food allergy and non-allergic food hypersensitivity, collectively known as food intolerance that includes metabolic disorders such as, lactose intolerance, phenylketonuria, or favism [4]. 

There is a global concern about the impact of food allergy on public health but also on its social and economic effects. Different patterns of food allergy have been identified in distinct ethnic groups and cultures, although the responsible factors are not fully understood [1]. According to Siecherer and Sampson, this problem affects nearly 8% of children and 5% of adults in developed Western countries, with growing evidence of an increase in prevalence due to the complexity of modern food and globalization [4]. The problem is also of particular concern in less developed countries due to the lack of public awareness and governmental regulation. 

The European Academy of Allergy and Clinical Immunology (EAACI) classifies adverse reactions to food into toxic and non-toxic [5]. Toxic reactions arise from the presence of toxins that may be naturally present in food, induced during processing, incorporated as an additive or by contamination. These toxins will cause a primary adverse effect on all individuals who consume them. Alternatively, non-toxic reactions are those that take place after the intake of foodstuff that is not tolerated by a few individuals. They can be sub-divided into immune-mediated adverse food reactions (food allergy) and non-immunological (food intolerance).

According to the National Institute of Allergy and Infectious Diseases (NIAID) sponsored guidelines, immune-mediated adverse food reactions can be classified into three categories [3,6]: 

(1) The so-called immunoglobulin E (IgE) antibody-mediated allergic reaction (immediate hypersensitivity). This allergic reaction is the most common source of allergy and the most widely studied. IgE-mediated food allergy involves the formation of IgE antibodies that recognize specific allergens in the food responsible for the allergy. There is a proven relationship between food intake and occurrence of symptoms. The severity of reactions, including the risk of anaphylaxis, is unpredictable and can be very different from one patient to another. Well-known allergens found in eggs, milk or peanuts are responsible for triggering IgE-mediated reactions.

(2) Non-IgE-mediated reaction (delayed hypersensitivity) are caused by a reaction involving other components of the immune system apart from IgE antibodies. For example: (a) a reaction involving antibodies of different isotypes from IgE (i.e., IgG, IgM or IgA); (b) a reaction depending on immune complexes formed by food allergens and antibodies; (c) cell-mediated immunity. Symptoms are related to the gastrointestinal tract as well as to the cutaneous and respiratory systems. The most common non-IgE-mediated reaction is celiac disease, where patients are sensitive to gliadin and suffer from malabsorption, chronic diarrhoea or abdominal distension.

(3) A combination of IgE mediated and non-IgE-reactions. 

Food intolerances cannot be considered as food allergies, but they show similar effects. They can be categorized as [1,4,7]: 

(a) Pharmacological, when they are caused by substances that show pharmacological activity, for example, vasoactive amines (dopamine, histamine, norepinephrine, phenylethylamine, serotonin and tyramine) and other substances present in foodstuff.

(b) Enzymatic, due to enzymatic defects in the gastrointestinal tract, for example, a deficiency of β-galactosidase that prevents the complete hydrolysis of lactose.

(c) Undefined intolerance, when the mechanisms of the intolerance have not yet been defined. However, some individuals show intolerance reactions to food additives, sulphites, nitrites, nitrates, monosodium glutamate and some colourings. The main symptoms may include asthma, rhinitis, urticaria, itchiness, and migraines, for example, gustatory rhinitis caused by the intake of spicy food which results in rhinorrhoea.

There are two main potential sources of allergens in food. The first includes food allergens that, with some exceptions, must be labelled. The second, compounds whose presence is the result of unintentional contamination, for example, because of the use of shared equipment or processing lines; inappropriate use of food processing aids or due to the lack of experience or law ignorance by the producer [8]. However, they are a real threat to the health of allergic persons. 

Very tiny amounts of allergens can be responsible for an allergic reaction in a sensitized consumer [9]. The symptoms may range from mild (itching, rashes, etc.) to severe (vomiting, diarrhoea, wheezing, anaphylaxis) and in extreme situations, they might even cause death. There is still no cure for food allergy, although some treatments are under study [9]. The only way to prevent it is to avoid allergen intake. Therefore, the food industry must provide precise and accurate information about the presence of allergenic ingredients in the labels of their products. 

Risk management of food allergens requires a safety policy consisting of three aspects: evaluation, management, and communication [10]. According to a report published in 1995 by the Food and Agriculture Organization of the United Nations (FAO) [10], eight allergenic foods, including milk, eggs, fish, crustaceans, peanuts, soybeans, tree nuts and gluten-containing cereals, were responsible for more than 90% of all food allergies. Therefore, foods containing those ingredients must be labelled as stated in the Codex Alimentarius (1999) [11]. However, the situation slightly varies in different countries. In North America, 0.6% of adults suffer from peanut and tree nut allergy, while 0.3% and 0.4% suffer from milk and fish allergy, respectively [9]. Cow’s milk is the most common source followed by egg (1.5%) and peanut (1%), although approximately 80% of these allergies are usually resolved by school age [9].

In the United States, food manufacturers must identify the presence of any of the main eight major allergens on their product labels, as stated by the Food Allergen Labelling and Consumer Protection Act of 2004 (FALCPA) [12]. Other allergenic foods do not need to be declared according to this law. Other countries, however, have an expanded list of major allergens. 

Directive 2000/13 (Commission of the European Communities, 2000) initially regulated labelling, presentation and advertising of foodstuffs produced in the Member States [13]. In order to guarantee a higher level of health protection for sensitized consumers Directive 2000/13 was amended by the Directive 2003/89 as regards indication of the ingredients present in foodstuffs. Directive 2003/89 [14] included in Annex IIIa a list of twelve ingredients (Table 1) that had to be labelled regardless of their concentration. According to this new legislation, “the list in Annex IIIa shall be systematically re-examined and, where necessary, updated by the most recent scientific knowledge [*omissis*].” It also established that “updating could also be affected by the deletion from Annex IIIa of ingredients for which it has been scientifically established that it is not possible for them to cause adverse reactions [*omissis*].” To that aim, the Commission shall, not later than 25 November 2004, after consultation with the European Food Safety Authority (EFSA), adopt a list of those ingredients or substances, which shall consequently be excluded from Annex IIIa, pending the final results of the notified studies, or at the latest until 25 November 2007.”

After consultation with EFSA, a new Directive 2005/25 (Commission of the European Communities, 2005, [15]) was adopted excluding some ingredients from the list in Annex IIIa of Directive 2003/89. In 2007, Annex IIIa was updated, and Commission Directive 2007/68 included new products in the list that become more restrictive (Table 1) [16]. Regulation (EU) No. 1169/2011 on the provision of food information to consumers brought together EU rules on general food labelling (Table 1) and nutrition labelling into one piece of legislation [17]. Implementation was mostly from 13 December 2014 although compulsory nutrition labelling was only applicable from 13 December 2016. Commission Delegated Regulation (EU) No. 78/2014 of 22 November 2013 amended Annexes II and III to Regulation (EU) No 1169/2011 of the European Parliament and of the Council on the provision of food information to consumers, as regards certain cereals causing allergies or intolerances and foodstuff with added phytosterols, phytosterol esters, phytosterols or phytosterol esters [18].

On July 2017, the Commission adopted a “Notice on the provision of food information to consumers on substances or products causing allergies or intolerances” [19]. This document updates the previous guidance document on allergen labelling issued under Directive 2000/13/EC. Its purpose is to assist consumers, businesses and national authorities in understanding the new requirements of Regulation (EU) No 1169/2011 related to the indication of the presence of certain substances or products causing allergies or intolerances.

In principle, the name of the allergen is composed of an abbreviation of the scientific name of its source (genus: 3–4 letters; species: 1–2 letters) and an Arabic numeral [20]. Information about the nomenclature, physicochemical properties and allergenic relevance of food allergens can be reviewed in several interesting databases [21]. For example, the official website of the World Health Organization and the International Union of Immunological Societies (WHO/IUIS) Subcommittee on Allergen Nomenclature (http://www.allergen.org/) [22], the Allergome database (http://www.allergome.org/) [23] and the All-Fam database (http://www.meduniwien.ac.at/allfam/) [24].

Plant allergens belong mostly to a few protein families (residue identities of 30% and greater) and superfamilies (low sequence identities but with structural and functional characteristics suggest a likely common evolutionary origin). Namely, the cupin superfamily (7S and 11S seed storage proteins), the prolamin superfamily (2S albumins, nonspecific lipid transfer proteins (nsLTPs), α-amylase/trypsin inhibitors, prolamin storage proteins of cereals), or the papain superfamily of cysteine proteinases (Table 2) [8,25,26]. Allergens from other plant protein families include Bet v 1-related proteins, profilins, kunitz-type protease inhibitors, lectins, patatin-like proteins, phenylcoumaran benzylic ether reductases, oleosins, β-fructofuranosidases, subtilisin-like serine proteases, flavin adenine dinucteotide containing oxides (Table 2). The variety of allergens in animal-derived food is smaller than in plants, and the most representative ones are shown in Table 3.

## 2. Current Analytical Methods for the Analysis of Food Allergens

Assessment of allergen labelling and risk management programs in food industry requires the availability of rapid, sensitive, cheap and accurate analytical techniques for the detection and quantification of allergenic residues in foodstuff. This can be a problematic analytical task because the concentration of these compounds in food matrices is usually low and matrix effects can mask their detection. Moreover, thermal or other technological treatments can modify the detection performance, together with the overall allergenicity of the food, hampering the development of reliable reference analysis methods [27].

Current methods for the detection of allergens in foodstuffs target either the allergen (protein) itself or a marker, such as specific proteins or DNA fragments, which indicates the presence of the food causing the allergy [9,28,29].

### 2.1. Protein Detection

Routine analysis of proteins in food is usually carried out by immunochemical techniques such as Western blotting and enzyme-linked immunosorbent assays (ELISAs) that provide semi-quantitative information [27,30]. Typical detection limits of ELISA are in the low ppm range (1–5 ppm), and some commercial kits allow qualitative analysis of common food allergens within short analysis times (30 min) without the need of skilled personnel [31]. 

The need for sensitive quantitative tests has also prompted the development of alternative fast and simple procedures based on lateral-flow immunochromatographic assays (LFA). Various food allergen detection kits based on immunochemical detection can be found on the market [32,33,34,35,36]. The main drawbacks of immunological methods are the specificity and stability of antibodies, as well as the cross-reactivity with the matrix components which may yield false positive or false negative results. On the other hand, antigen-antibody interactions can also be affected by changes induced in the proteins by food processing. 

### 2.2. Nucleic Acid Detection

The detection of species-specific DNA molecules is usually accomplished using polymerase chain reaction (PCR) amplification of DNA segments specific for the allergenic protein of interest. Information on methods of allergen detection using PCR reactions, namely PCR-ELISA, real-time PCR, PCR-peptide nucleic acid-high performance liquid chromatography, duplex PCR can be found in several reviews [9,28,37,38,39]. Nowadays, there is a great interest in multiplex approaches such as multiplex real-time PCR that allows simultaneous detection of several DNA fragments at the same time.

It must be borne in mind that the presence of a DNA fragment corresponding to the gene of a protein in a food commodity is not indicative of the presence of the allergen itself and vice versa [39]. On the other hand, as discussed previously, heating and food processing may affect the concentration of nucleic acids and proteins in different ways. Therefore, there is some controversy in the use of DNA methods for the analysis of allergens in place of immunological methods [9,28]. In the past years, mass spectrometry has become an essential tool for the identification and quantification of food allergens in different commodities [9,40,41,42]. The combination of several “-omic” techniques, namely proteomics, peptidomics and metabolomics, into a new discipline of foodomics has proven to be of great interest in the evaluation of food allergy, including the structural characterization of novel food allergens. The formation/fate of allergens along the technological processes or the evaluation of protein modifications in food for allergic patients are also task to assess.

Biosensors are a potential alternative to classical methodologies for direct, reproducible, real-time and cheap detection of food allergens. Sensors are defined as integrated receptor-transducer devices which provide specific quantitative or semi-quantitative analytical information on the species of interest [43]. Biosensors contain a biological recognition element (biochemical receptor) in direct spatial contact with a transducer, which converts the recognition event into a measurable chemical or physical signal, connected to data acquisition and processing system [27,43]. 

Recent advances in the area of material science and the combination of new selective bio- and biomimetic receptors with the unique optical, electrical, or electrochemical properties of nanomaterials have allowed the development of new nanosensors that could lead to significant improvements for example in food safety, quality control or traceability [44]. Some of the advantages of nanosensor applications for allergen detection include low detection limits (single molecule detection), reduction of reagent volumes, shorter analysis times or multianalyte detection among others [44]. 

In this review, we present some recent advances on developing novel biosensors based on nanoparticles (NPs) for the detection of food allergens based on different transduction principles (i.e., electrochemical, optical, etc.) and recognition elements (i.e., antibodies, oligonucleotides, aptamers, etc.).

## 3. Bioconjugation of Nanoparticles

The increasing availability of new technologies to manufacture and characterize novel materials at nano-scale has generated considerable interest in the field of biotechnology [45,46]. Certain nanomaterials exhibit particular properties that cannot be extrapolated from their bulk behaviour but result extremely advantageous for biosensor design. For example, their size, shape, functionality, surface area, together with their unique optical, electronic, or magnetic properties have enabled fabrication of more efficient biosensors and bioassays in the last years [46,47].

One of the advantages of the application of nanomaterials in biosensing is the possibility to combine them closely with the biological recognition element [48,49]. Nanostructures are suitable for the development of composite nanoscale materials in combination with biological entities, such as enzymes, proteins, antibodies, peptides, oligonucleotides (e.g., DNA; RNA, LNA, aptamers) or carbohydrates, which combine the functionalities of biomolecules with the tailor-controlled sensing characteristics of nanomaterials [50]. Among all the nanomaterials produced so far, the most widely applied for the detection of food allergens in combination with biosensors are: metal NPs (gold and silver), carbon nanomaterials (e.g., carbon nanotubes, graphene), magnetic NPs and semiconductor quantum dots [51,52].

**Figure 1:**
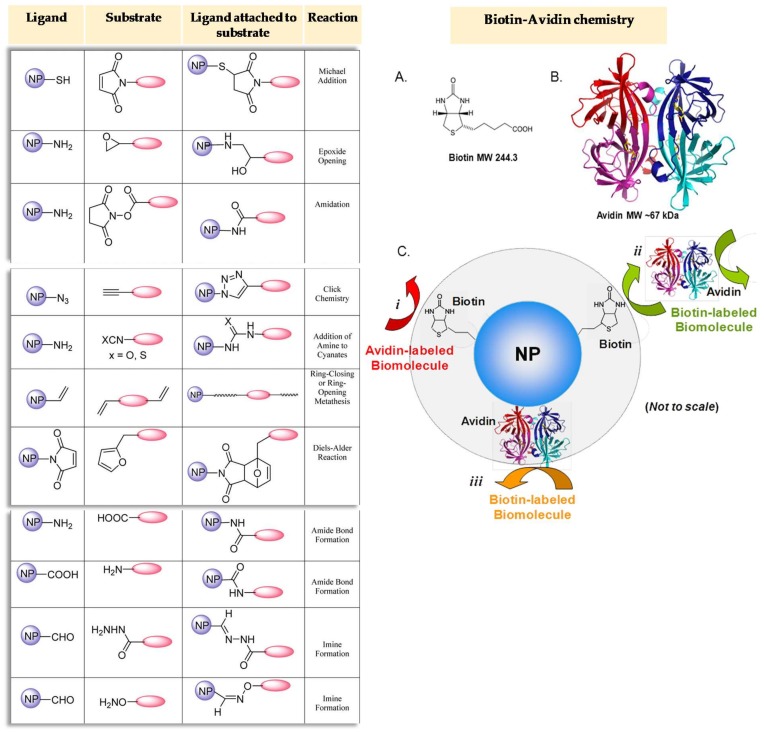
Selected nanoparticle (NP) functionalization chemistries. Biotin−avidin chemistry. (A) Chemical structure of biotin. (B) a ribbon model of tetrameric avidin, showing the monomers in magenta, blue, cyan and red and the four biotin molecules in yellow. (C) Scheme showing the three commonly used biotin−avidin strategies for attaching biomolecules to NPs: (i) Biotin-labelled NP coupled to avidin-labelled biomolecule; (ii) Biotin-labelled NP coupled to a biotin-labelled biomolecule by an avidin linker; (iii) Avidin-labelled NP coupled to a biotin-labelled biomolecule. Reprinted with permission from [50]. Copyright 2013 American Chemical Society.

Selection of the bioconjugation chemistry for biosensor development depends on several factors including the type of NP material, its size, shape, surface chemistry and structure, the type of ligand bound to the NP and the type of biological molecule [50]. Conjugation chemistries traditionally applied for the immobilization of biomolecules on different substrates are also applied in combination with nanomaterials. However, efforts are being focused on the search of new non-heterogeneous and site- or regiospecific chemistries that improve the performance of existing NP bioconjugate constructs [50]. Figure 1 shows some of the methods usually applied for the immobilization of biomolecules in nanomaterials for sensing purposes.

## 4. Applications of NPs in Bioassays and Biosensors for Food Allergen Detection

In the last ten years, the number of peer-reviewed contributions on food allergen detection combining bioconjugated NPs and biosensing platforms has steadily increased. For example, at the beginning of the 21st century, only 2% of publications on bioassays for allergen detection were based on the use of NPs. However, during the last 3 years, more than 40% of the reported bioassays are based on nanomaterials, although the number of contributions in this field is still limited.

### 4.1. Electrochemical Biosensing

Electrochemical biosensors and bioassays are widely applied to the analysis of food and beverages. They can be categorized as potentiometric, amperometric, voltammetric, conductometric or impedimetric sensors depending on the electrochemical principle involved [27]. Amperometric biosensors are probably the most widespread, numerous and most successfully commercialized devices based on biomolecular electronics [53]. Electrochemical biosensors may be advantageous for the analysis of allergens in complex food samples over other types of devices considering their cost, instrumental simplicity, ease of fabrication, sensitivity, linear response range, rapidity and portability. The incorporation of nanomaterials in sensor design holds the potential to improve the performance of existing devices either by improving the response of the transducer, by immobilizing the bioreceptor without compromising its biological activity, or by creating innovative alternatives for labelling, including the capability for multiplexing [54]. In this context, signal amplification has been attributed on the one hand to the increment in the loading of electrochemically detectable species by the NPs supported on the electrode surface [55]. On the other hand, modification of the electrodes with metallic NPs facilitates the flow of electrons (electrocatalysis) between the solid surface and adhering biomolecules [56], which improves sensor sensitivity. Several reviews have been published in the last years on the use of nanomaterials in electrochemical sensors and their applications to food analysis [55,57,58,59,60]. Table 4 summarizes the analytical characteristics of relevant electrochemical biosensors based on NPs for the detection of food allergens.

Gold nanoparticles (AuNPs) are the most widely used NPs for signal amplification since they can be synthetized in several sizes and formats, can be easily functionalized and are highly stable [55,61,62]. Several approaches have been reported in the literature on the application of AuNPs in combination with electrochemical biosensors for the analysis of food allergens. Qao et al. [62] developed a selective electrochemical immunosensor for the determination of casein—one of the major allergens of milk—in cheese samples based on the combination of AuNPs and poly(l-Arginine)/multiwalled carbon nanotubes (P-l-Arg/MWCNTs) in a composite film. A glassy carbon electrode (GCE) was modified with the P-l-Arg/MWCNTs film through the electropolymerization of l-Arginine on MWCNTs/GCE. AuNPs were adsorbed onto the surface of the modified electrode by chemisorptions interactions with the NH_2_ group of P-1-Arg and further functionalized with the anti-casein antibody. This approach increased the conductibility, biocompatibility and the amount of antibody immobilized onto the electrode surface. Casein detection was monitored by cyclic voltammetry (CV) and differential pulse stripping voltammetry (DPV) using a ferricyanide probe. The sensor exhibited a lower detection limit (5 × 10^−8^ g/mL) and a wider response range than other methods described in the literature for the analysis of casein in cheese, based for example on ELISA or surface plasmon resonance (SPR) measurements. The improved sensitivity was attributed to both the excellent electrical conductivity of MWCNTs and the high amount of AuNPs immobilized to P-l-Arg with the corresponding increase in the surface loading density of anti-casein antibody. 

**Figure 2:**
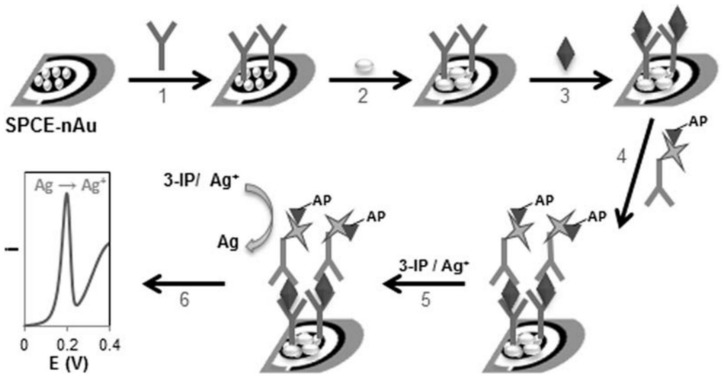
Schematic representation of the construction of the sensor surface and the immunoassay. (1) Capture antibody immobilization, (2) surface blocking (casein), (3) antigen (standard/sample) addition, (4) detection antibody–S-AP addition, (5) substrate (3-IP) and silver ions addition and (6) voltammetric detection of Ag^0^. Reprinted with permission from [63]. Copyright 2015 Elsevier.

In an alternative approach, Alves et al. [63] reported the development of a voltammetric immunosensor for the detection of Ara h 1, a major peanut allergen, using screen-printed carbon electrode (SPCE) coated with AuNPs (Figure 2). Metal NPs were directly generated on the surface of the SPCE through electrochemical deposition of ionic gold. The sensing strategy was based on a sandwich-type immunoassay using two monoclonal mouse anti-Ara h 1 IgGs as capture and detection antibodies. The first was immobilized on the Au-nanostructured surface while the detection antibody was labelled with the enzyme alkaline phosphatase. Metallic silver (Ag) was obtained in the course of the enzymatic reaction and linear sweep voltammetry monitored the electrochemical stripping current of the enzymatically deposited silver. The immunosensor was successfully applied to the analysis of peanut residues in cookies and chocolate. The relatively long response time (3 h 5 min) was the main drawback of this biosensor in comparison with others reported in the literature. Although the calibration range (25–2000 ng/mL) was wider and the detection limit significantly lower (3.8 ng/mL) than with other antibodies [64,65] or DNA-based biosensors [66] for Ara h 1. These authors followed the same strategy for the analysis of the Ara h 6 allergen, in the same samples, with a limit of detection of 0.27 ng/mL [63].

**Figure 3:**
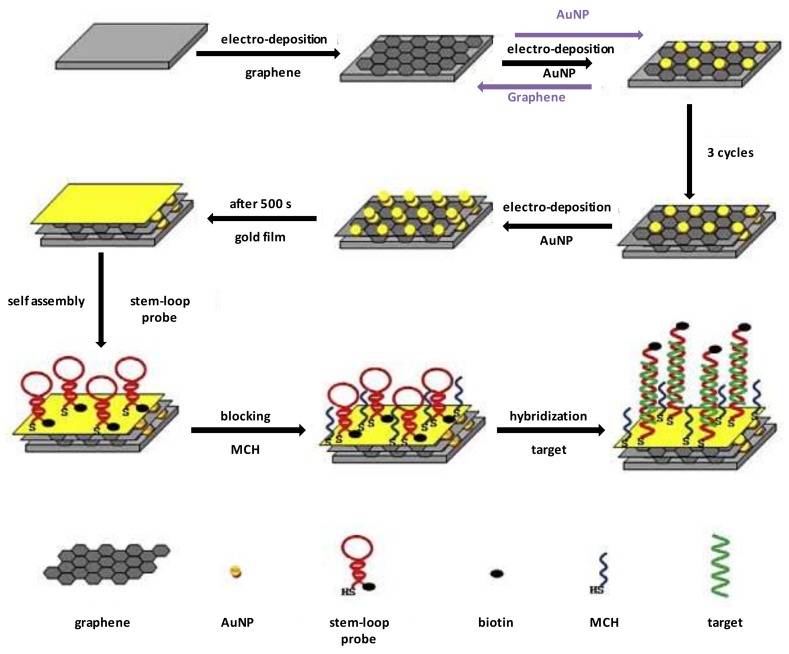
Schematic illustration of the fabrication of the electrochemical stem-loop DNA biosensor. Reprinted with permission from [68]. Copyright 2012 American Chemical Society.

Liu et al. [67] developed electrochemical immunosensors for the detection of antibodies against Ara h 2, using glutathione-decorated AuNPs (GSH-AuNP) which were functionalized with 28 amino acid peptide fragments of the major IgE-binding epitope of Ara h 2 and coated on the surface of pyrolytic graphite electrodes. They compared the performance of different detection methods namely, single label amperometry, faradaic electrochemical impedance spectroscopy (EIS) and non-faradaic impedance for the detection of model chicken anti-peanut antibodies (IgY) in serum. The best detection limits (5 pg/mL) and linear range (from 5 pg/mL to 1 μg/mL) were obtained with the sensor measured by non-faradaic impedance with horseradish peroxidase (HRP) amplification. Alternatively, the analysis of Ara h 1 allergen gene was carried out using DNA-based sensors. The biosensor reported by Sun et al. [68] was based on the cyclic electrodeposition of alternate monolayers of graphene and AuNPs on the surface of a GCE to obtain a multilayer graphene–gold nanocomposite. Next, a gold layer was electrodeposited on the surface of the nanocomposite multilayer and stem-loop probes, dually labelled with 5′-SH and 3′-biotin, were assembled on the gold film/graphene–gold nanocomposite/GCE surface, shown in Figure 3. Hybridization of the probes with the target DNA sequence opened the stem-loop structures resulting in a decrease of the peak current and an increase of the impedance that correlated with the concentration of allergen in the sample. The sensor showed a detection limit of 4.1 × 10^−17^ M and excellent selectivity for the analysis of Ara h 1 gene in peanut milk beverages. 

The same group prepared Ara h 1 biosensors [69] using chitosan-multiwalled carbon nanotube nanocomposite (CS-MWCNT) coated on GCE, followed by a spongy gold film via electro-deposition. Biotinylated stem-loop probes were immobilized on this platform and hybridization with the target DNA triggered the specific interaction between biotin and streptavidin bound to HRP. The concentration of allergen in the sample was electrochemically evaluated by monitoring the enzymatic product in the presence of a substrate. The application of this approach allowed a slightly lower detection limit than the multilayer graphene–gold nanocomposite (1.3 × 10^−17^ mol/L).

A disposable amperometric biosensor based on AuNP-modified screen-printed carbon electrodes was developed by Manfredi et al. [70] for fast analysis of celiotoxic prolamins (gliadin from wheat). Gliadin was immobilized on the electrode surface and the sensing was based on a competitive immunoassay with a polyclonal rabbit anti-gliadin antibody. Alkaline phosphatase conjugated anti-rabbit IgG was used as the secondary antibody to monitor the concentration of gliadin in cereal-based food samples. The biosensor showed a detection limit of 8 ng/mL of gliadin in ethanol extracts and it could be a useful tool for the safety assessment of raw materials used in the production of dietary products for celiac patients. 

In an alternative approach, Jiang et al. [71] developed an electrochemical rat basophilic leukaemia cell (RBL-2H3) sensor for the determination of allergens in food using fluorescent magnetic beads. The authors entrapped fluorescein isothiocyanate (FITC) in the SiO_2_ shell of Fe_3_O_4_@SiO_2_ core-shell nanoparticles that were further encapsulated with a liposome to form cationic magnetic fluorescent nanoparticles (CMFNPs) for mast cell magnetofection. The CMFNPs-transfected RBL-2H3 cells activated by an allergen antigen were adsorbed to the surface of a magnetic glassy carbon electrode (MGCE) for analysis and a dose depending correlation between the electrochemical impedance signal and the target concentration was obtained. The sensor was applied to the detection of shrimp allergen tropomyosin (Pen a 1) and fish allergen parvalbumin (PV) using anti-Pen a1 IgE and anti-PV IgE-activated cells, with a limit of detection of 0.03 μg/mL and 0.16 ng/mL, respectively.

### 4.2. Optical Biosensing

Biosensors for food analysis commonly rely on optical or electrochemical signal transduction techniques. Optical transducers transform the variations in an optical phenomenon, originating from the interaction between the analyte and the bio-recognition element, into an electrical signal [76]. Optical biosensors usually offer high specificity, sensitivity, small size and cost-effectiveness. In comparison with traditional analytical techniques, they enable direct, real-time and label-free detection of many biological and chemical substances. These devices can also be adapted to remote sensing, multianalyte detection and are easily miniaturized, allowing their integration into more complex systems [77,78]. Optical biosensors can be broadly divided into two sensing modes: (a) label-free sensors where the detected signal is generated directly by the interaction of the analyte with the transducer and, (b) label-based sensors that require the use of a label and the analyte-receptor binding event is transformed into a measurable absorption or luminescent signal [79].

The most commonly used transduction techniques in optical biosensors based on NPs for food analysis are UV-vis and infrared absorption, fluorescence, chemiluminescence, surface plasmon resonance (SPR), surface-enhanced Raman scattering (SERS) and interferometry. The analytical characteristics of selected optical biosensing approaches based on NPs for the detection of allergens in food are summarized in Table 5 and Table 6.

#### 4.2.1. Colorimetric Detection

Colorimetric detection is simple, fast, highly sensitive and selective and represents a versatile tool for sensor development. It measures the amount of light absorbed by chromogenic reagent or a reaction product at a selected wavelength characteristic of the selected reagent. The absorbed light will be proportional to the concentration of the reagent in the sample [80]. Detection can be carried out without complex instrumentation, or even with a naked-eye and nowadays the use of smart-phones for this application has become very popular [81].

Plasmonic metal nanomaterials (PMNMs), especially gold and silver, play an important role in the development of novel colour-based biosensors for allergen detection. These metals show a particular phenomenon known as localized surface plasmon resonance (LSPR) produced by the collective oscillation of free electrons in the presence of light with particular wavelength [82,83]. Any variation in the presence of the target compound in the shape, size, geometry, surface capping, medium refractive index, or the dispersion state of NPs, will affect the local electron confinement causing a variation of the SPR absorption maxima and in the colour of the colloidal solution [83]. 

Several immunoassays based on the use of PMNMs and colorimetric detection have been reported for the rapid detection with simple operation, low cost, visual results and high sensitivity to allergens in food [43,84]. In most cases, they are based on lateral flow biosensors (LFBs) which are easy-to-use devices combining the advantages of lateral flow devices (LFA) such as portability, low-cost and rapidity, with the specificity and selectivity of immunoassays [85,86].

Gold immunochromatographic lateral flow assays (GICAs) usually provide semi-quantitative, or just qualitative results and are based on a sandwich assay format. For example, Zhigang et al. [87] developed a portable GICA for the detection of milk allergen β-lactoglobulin (βLG). The sensor was based on a sandwich assay format using two monoclonal antibodies, mAb 1H8 conjugated to AuNPs and mAb 1G5 immobilized on the detection line. Upon target binding, the sandwich complex with the AuNP-labelled antibody was formed in the detection line giving a positive visually red assay response. The GICA assay performance was compared with a traditional ELISA, using the same monoclonal antibodies and the sensitivity in milk extracts was significantly improved (detection limits of 0.8 ng/mL for ELISA and 0.2 ng/mL for GICA). Moreover, no cross-reactivity was observed towards other possible interfering species such as BSA, caseins or α-lactalbumin. The method was successfully applied to the detection of βLG in several food samples (peanut, egg, fish, shrimp, cashew, wheat and soybean).

**Figure 4:**
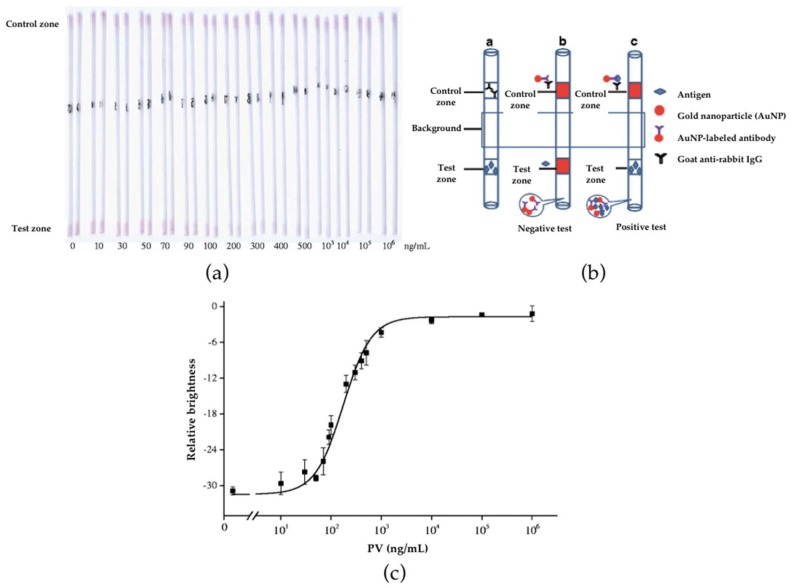
(**a**) Colloidal-gold immunochromatography assay in modified glass capillaries (CICA) results for parvalbumin (PV) in phosphate-buffered saline (PBS). PV concentrations tested ranging 0 to 106 ng/mL; (**b**) scheme of CICA system and (**c**) calibration curve obtained (n = 3). Reprinted with permission from [88]. Copyright 2014 Springer.

In a different approach, a colloidal-gold immunochromatography assay in modified glass capillaries (CICA) was proposed by Cao et al. [88] for the detection of parvalbumin (PV). Based on a direct competitive immunoassay, both PV and goat anti-rabbit IgG were immobilized inside glass capillaries in the test line and in the control line, respectively. As shown in Figure 4, in the presence of PV in the sample, the free analyte competed with the immobilized allergen for binding to the AuNP-labelled anti-PV conjugate and no signal was observed in the test zone. However, a red band was observed in the control zone due to the accumulation of the AuNP-labelled antibodies.

The developed CICA assay showed a visual detection limit (VDL) of 70 ng/mL and a semi-quantitative limit of detection, measured with a flatbed scanner, of 40 ng/mL. The authors suggested that the sensitivity is sufficient for efficient screening of PV in foodstuffs although the approach was less sensitive than previously reported ELISAs [89,90]. The sensor exhibited good reproducibility (CV% ranges 2.7–9.7%) and cross-reactivity was negligible for human serum albumin and egg albumin. This approach featured better stability (4 weeks, 4 °C) than GICA methods performed on nitrocellulose membranes. The immunosensor was successfully applied to the analysis of PV residues in several samples, including surimi products, livestock, confirming sufficient accuracy and precision of the proposed CICA method. According to the authors, the CICA system could be used for routine analysis of the PV fish allergen.

**Figure 5:**
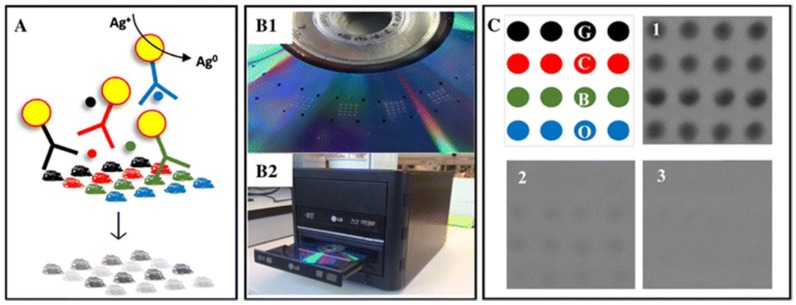
Scheme of a competitive microimmunoassay on DVD. (**A**) The protein allergens (gliadin, casein, β-lactoglobulin and ovalbumin) are immobilized on the DVD surface. AuNPs-labelled specific antibodies bind either to the specific immobilized allergens or to the analytes in solution (allergens). The antibody-antigen interaction is displayed as a black precipitate (grayscale) after Ag enhancement step. Each array (**B1**) is localized by four marks made by a waterproof pen and the disc is scanned by the DVD drive (**B2**). (**C**) Data analysis: panels 1–3 correspond to the images of the arrays for allergen concentrations of 0, 1.25 and 10 mg/L, respectively. Reprinted with permission from [91]. Copyright 2017 Springer.

Optical microarrays are a very promising tool for the analysis of food allergens in the food industry as they allow simultaneous detection of several allergens and food residues using simple, easy to operate and low-cost instrumentation. Maquieira and co-workers [91] developed a multiplex competitive microimmunoassay for the simultaneous detection of four food allergens including gliadin, casein, β-lactoglobulin and ovalbumin using Digital Versatile Discs (DVD) as sensing platforms. Protein allergens were immobilized at the desired positions on a polycarbonate DVD using a non-contact printer (AD 1500 BioDot, Inc., Irvine, CA, USA). The affinity interaction between the immobilized allergen and its respective AuNP-labelled specific antibody was detected by measuring the optical density of a precipitate formed by the oxidation of the AuNPs with an Ag enhancer solution. The response was proportional to the concentration of allergen in the sample (Figure 5). The sensitivity of the multiplexed assay, measured as EC_50_, was 0.04, 0.40, 0.08 and 0.16 mg/L for gliadin, casein, β-lactoglobulin and ovalbumin, respectively. Cross-reactivity studies reported a slight interference (2.0%) of casein and minimal interference of ovalbumin (<0.36%), in the gliadin assay. The authors point out that the assay format is critical to set-up a practical multiplex assay, as the sandwich format (coupled to an enzymatic detection approach) was more sensitive in most cases. However, the competitive format is more functional from the DVD-based fabrication point of view.

#### 4.2.2. Fluorimetric Detection

Fluorescence is one of the most popular detection methods used in biosensing. Several parameters can be determined for sensing purposes including light intensity, decay time, or emission anisotropy [43,94]. This technique is especially suitable when meagre detection limits are essential and nowadays fluorescence transduction can be implemented using relatively simple schemes without the need of sophisticated instrumentation [95]. Furthermore, these systems can be easily adapted to simultaneous detection of multiple allergens, which is of particular interest when some of them are found in the same foodstuff [96].

One of the significant difficulties for the development of fluorescent biosensors is the synthesis of labelled derivatives that simultaneously combine a specific affinity for the bioreceptor, adequate solubility in water, suitable affinity constants and satisfactory photochemical performance to report the binding event with sufficient sensitivity [97]. On the other hand, conjugation of fluorescent dyes to bioreceptors can be very difficult and can affect their biological activity negatively. The application of nanomaterials overcame some of these limitations allowing the implementation of new ultra-sensitive detection schemes with improved functionalities such as excellent absorption capacity of the bioelements involved in the detection, minimization of non-specific binding, improvement of mass transfer and implementation of unique optical properties [98].

Quantum dots (QDs) are the most widely applied NPs in fluorescence biosensing [99]. They feature high photoluminescence quantum yields, size-tunable emission acquired in a narrow and symmetrical spectrum, broad absorption spectra and excellent resistance to photobleaching [52]. The main disadvantage of QDs is their high toxicity, as they are made up of heavy metals. However, in recent years several coatings have been implemented to avoid this risk and to improve their compatibility with aqueous media [100].

**Figure 6:**
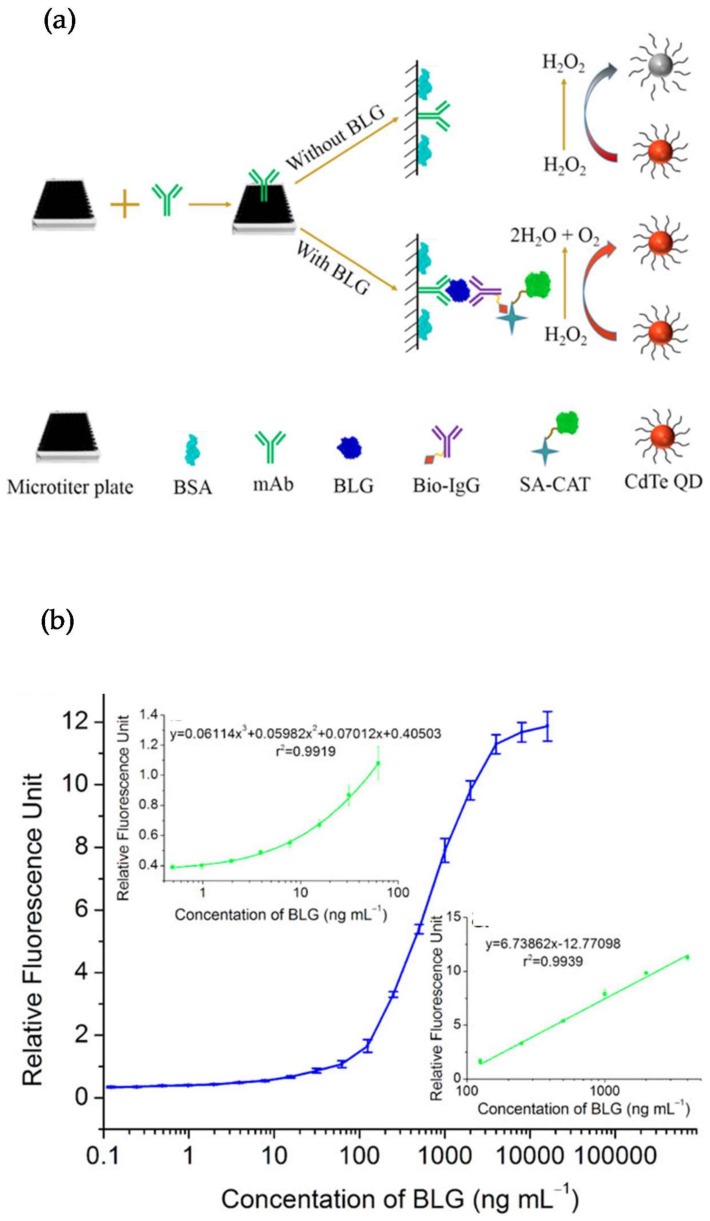
(**a**) Schematic principle of the immunoassay based on catalase-mediated fluorescence quenching of H_2_O_2_-sensitive quantum dots (QDs) for the detection of β-La; (**b**) Calibration curve of fluorescent ELISA for detection of β-La. Reprinted with permission from [102]. Copyright 2018 Wiley.

Several approaches have been reported in the literature describing the application of QDs for the analysis of food allergens. Yang et al. [101] developed an immunoassay for the detection of α-lactoglobulin (α-La) in commercial dairy products using monoclonal antibodies against α-La covalently conjugated to CdSe/ZnS QDs. The new approach, known as fluorescent-linked immunosorbent assay (FLISA), was compared to a conventional competitive inhibition ELISA. The best limit of detection (0.1 ng/mL) and wide dynamic range (0.1–1000 ng/mL) were obtained using FLISA. In an alternative approach, He et al. [102] detected the allergen β-lactoglobulin (β-La) using catalase-mediated fluorescence quenching of thiolated CdTe quantum dots in the presence of H_2_O_2_ as fluorescent signal output [103,104]. The primary monoclonal antibody (1G9), specific to β-La, was immobilized on a microtiter plate and the sensing mechanism was based on a sandwich assay format using biotinylated rabbit anti-β-La polyclonal Igs as secondary antibodies. Catalase conjugated to streptavidin was bound to the secondary antibody and its catalytic activity prevented the oxidation of the thiolated CdTe quantum dots by H_2_O_2_ in the presence of β-La in the sample (Figure 6a). The bioassay showed a detection limit of 0.49 ng/mL, which was a 16-fold lower than the reference ELISA based on HRP (Figure 6b). The method was applied to the analysis of β-La in real samples such as UHT milk, whey, wheat, cookies and infant foods, with recoveries ranging from 94.25 to 109.83%.

**Figure 7:**
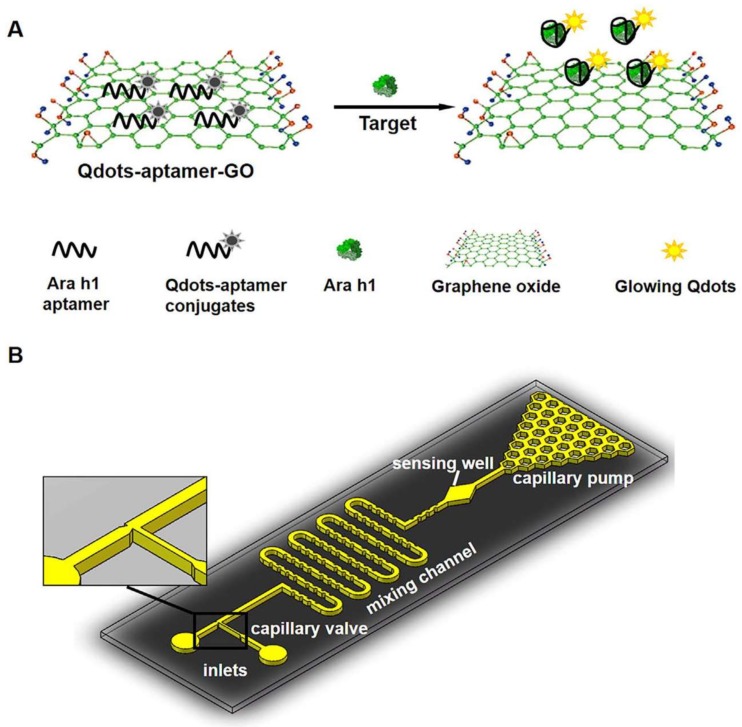
(**A**) Schematic of the sensing mechanism of the QDs-aptamer-GO quenching system; (**B**) Schematic diagram of microfluidic chip design (not to scale). The chip had two inlets for loading the QDs-aptamer-GO probe mixture and the Ara h1 sample, respectively. The main channel consisted of a mixing/incubation channel, a sensing well and a capillary pump at the end. The “diamond”-shaped well was the sensing well aligned to the sensing window of the Si photodiode. Reprinted with permission from [107]. Copyright 2018 Wiley.

One of the most used strategies in fluorescence biosensing for the detection of allergens is based on the use of noble metals and carbon nanostructures, such as AuNPs or graphene oxide (GO), combined with aptamers [105]. In most cases, the sensing principle is based on the fluorescence quenching of the aptamer conjugates absorbed by π-π stacking interactions onto the nanostructure surface. For example, Zhang et al. [106] fabricated a sensitive biosensor for the detection of shrimp allergen tropomyosin by immobilizing a selective aptamer on a GO substrate, by hydrophobic and van der Waals interactions. Addition of tropomyosin resulted in a change of the aptamer shape and subsequent release from the GO surface. The aptamer-tropomyosin complex in solution was detected by fluorescence spectroscopy (λ_em_ = 480 nm; λ_em_ = 522 nm) in the presence of the OliGreen ssDNA reagent. The application of this approach allowed a detection limit of 4.2 nM and a dynamic range of 0.5–50 μg/mL with negligible cross-reactivity towards BSA, streptavidin, lysozyme, thrombin, β-conglycinin and myosin. In an alternative approach, Weng et al. [107] developed an integrated microfluidic platform with aptamer/Qdots-functionalized GO for Ara h 1 detection. The sensing mechanism relied on the fluorescence resonance energy transfer between the Qdots-aptamer and GO. In the presence of the peanut allergen, the signal is “turned on” due to the release of the Qdots-aptamer-Ara h 1 complexes from the GO substrate with the corresponding increase in the fluorescence signal measured in the microfluidic chip coupled to a high-sensitive Si photodiode (λ_em_ = 531 nm) (Figure 7). The limit of detection was 56 ng/mL, the dynamic range 200–2000 ng/mL and no cross-reactivity was observed with Ara h 2 and Ara h 3 proteins.

#### 4.2.3. Surface Plasmon Resonance Detection

Surface plasmon resonance (SPR) spectroscopy is a label-free technique used for decades in the development of optical sensors and biosensing systems [94]. Among other advantages, it allows the determination of kinetic and affinity constants as well as real-time quantification of an analyte by detecting its binding to a receptor immobilized on the sensing surface [108]. Surface plasmons are collective oscillations of metal electrons that can be excited by a polarized light beam at a specific angle of incidence, called resonance angle, at the interface between two media with different dielectric constants, that is, a metal and a dielectric [109]. The excitation of SPR has been done using several methods, including angle, wavelength, intensity and phase interrogation techniques [110]. The number of publications of SPR-based methods for the analysis of food allergens is remarkable [27,111,112] however, there are not many examples in combination with NPs [17,113].

**Figure 8:**
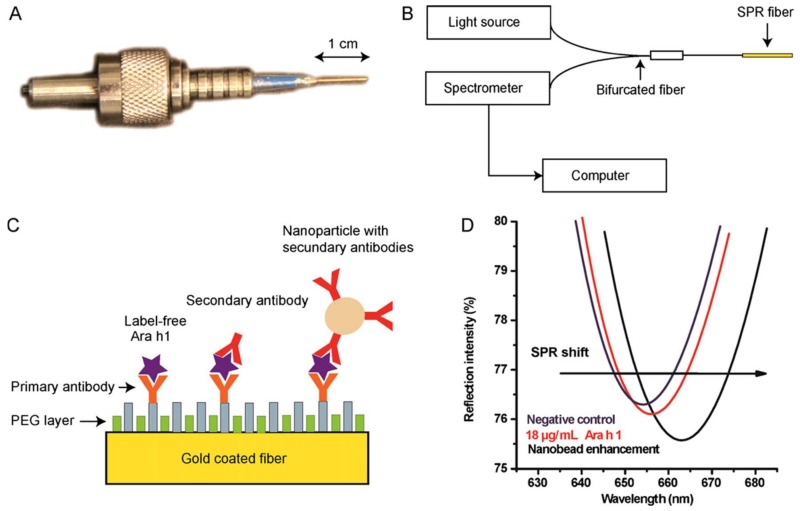
(**A**) Fibre optic surface plasmon resonance (SPR) probe; (**B**) system setup scheme; (**C**) Ara h 1 immunoassay detection strategies on the fibre optic SPR biosensor; (**D**) the spectrum dips in PBS buffer after 10 min incubation of the SPR fibre in: a negative control sample (blue dip), a sample containing 18 μg/mL Ara h 1 (red dip), a sample containing 18 μg/mL Ara h 1 subsequently labelled with antibody linked nanobeads (black dip). Reprinted with permission from [65]. Copyright 2018 Wiley.

Pollet et al. [65] described the first fibre optic SPR biosensor, with a nanobead enhanced SPR signal, to detect Ara h 1 in food matrices (Figure 8). The authors compared three different immunosensing strategies for further sensor development using this platform: a label-free assay, a sandwich assay using a secondary antibody and a nanobead-enhanced sandwich assay. They found that the use of 19 nm-size superparamagnetic NPs coated with the detection antibody, improved the detection limit of the SPR sensor two orders of magnitude, from 9 to 0.09 μg/mL, in comparison with the other approaches and no loss of sensitivity was observed after 35 regeneration cycles. The authors applied the method to the detection of Ara h 1 in chocolate candy bars and compared the results with those obtained with a commercial ELISA kit. The correlation between both methods was excellent but the sensor showed a more extensive dynamic range (0.1–2 μg/mL). The versatility of this biosensing platform has been demonstrated by the same research group who developed a fibre optic SPR aptasensor for the determination of Ara h 1, both in buffer and food matrices, using AuNPs amplification [114].

When the dimensions of noble metal nanostructures are lower than the wavelength of the incident light, the electronic oscillations are confined within a small volume, resulting in a particular type of surface plasmon called localized surface plasmon (LSPR). LSPR is strongly dependent on the refractive index of the surrounding medium and the morphology of the metal nanoparticle [115]. There are many examples in the literature using LSPR transduction for biosensor development, however, only a few are focused on allergen detection. For instance, Minh Hiep et al. [116] reported an LSPR-based immunosensor for label-free detection of casein in raw milk samples. The authors synthesized a gold capped silica nanoparticle substrate decorated with anti-casein antibodies immobilized in an oriented way using protein A. Under optimal conditions, the biosensor showed a detection limit of 10 ng/mL and a linear response range between 0.1 and 10 μg/mL.

LSPR also concentrates the electromagnetic (EM) field around the nanostructure and the local EM field leads to an electric dipole in the NPs that can influence the optical processes, such as absorbance, fluorescence or Raman scattering [115]. This observation has given rise to different spectroscopic techniques such as resonance-enhanced absorption (REA) [117], surface-enhanced fluorescence (SEF) [118] and surface-enhanced Raman scattering (SERS) [119,120]. Maier et al. [84] reported the development of an optical immunochip using AuNPs as signal transducers in a highly sensitive interferometric setup. The biosensor was applied to the analysis of ovalbumin (OVA) and ovomucoid (OVO) based on REA and in a direct and two-site sandwich-type immunoassay format, respectively. They created a multilayered resonant system using a highly reflective aluminium disk, an optically transparent polymeric distance layer and a bio-recognitively bound metal nanocluster layer for colorimetric readout. The surface of the polymeric distance layer was spotted with OVA and OVO for the direct assay format. A blue resonance colour became visible in the spotted area when AuNP-conjugated anti-OVA, or anti-OVO IgGs, were bound to the immobilized antigen as they were located within the essential resonance distance. Alternatively, in the sandwich assay format, the chips were first coated with the antibodies. The assay was semi quantitative and visible to the naked eye. The limit of detection was 1 ng/mL and dynamic range between 1 ng/mL and 1 mg/mL. The immunosensor was used for the analysis of egg-based foods, demonstrating its applicability in complex matrices. Nevertheless, authors suggested the need for further investigations to improve the performance in the micromolar range as well as to avoid false-positive results due to the unspecific interaction of Au clusters with matrix compounds.

In a different approach, Xiaoyan et al. [121] developed a SERS-based DNA-biosensing strategy for the detection of Ara h 1 using bipyramid-shaped gold nanocrystals (BPGNs), obtained by a novel synthesis protocol. The NPs were generated using sodium dodecylbenzene sulfonate (SDBS) which accelerates the side growth over twin boundaries. The sensing strategy was based on the use of a Cy3-tagged molecular beacon, which was specific to Ara h 1 gene and thiol-modified at 5′-end for immobilization onto the surface of BPGNs. Hybridization with the target DNA sequence opened the stem-loop structure of the molecular beacon resulting in a decrease of SERS signal because of the separation of Cy3, at the 3′-end, from the surface of the BPGN. The assay showed a limit of detection of 3.3 × 10^−15^ M and a linear range from 10^−14^ to 10^−8^ M. The platform was used for the determination of Ara h 1 in peanut by-products. Similarly, Boushell et al. [122] reported a SERS biosensing system for monitoring lysozyme using dendritic silver nanoparticles (AgNPs). The assay format was based on the use of an aptamer highly specific to egg white lysozyme coupled to the NPs. The SERS signal was a combination of both the lysozyme signal and the aptamer signal thus, a principal component analysis method was applied for quantification. The lowest detectable concentration for lysozyme in water was 0.5 g/mL. This value increased to 5 g/mL in stainless steel food-handling surfaces.

### 4.3. Others

Piezoelectric sensors based on the use of quartz crystal microbalance (QCM) have also been applied to the detection of allergens in food. Biosensor development requires immobilization of the selective antibody, alternatively, the antigen on the piezoelectric sensing platform. Xiulan et al. [74] applied 1,6-hexanedithiol/nanogold-modified composite architectures for the analysis of shrimp allergens. This approach allowed increasing the amount of immobilized anti-shrimp antibodies, while preserving their activity and the subsequent allergen mass on the QCM chip. Moreover, the use of NPs allowed a 3D orientation of the biomolecules increasing the likelihood of the antigen-antibody interaction and thus improved the sensitivity of the immunoassay. The shrimp antigen was detected directly by monitoring the decrease in the resonance frequency due to the binding of allergen onto the immobilized antibodies. The device showed a fast response time (10 min), a detection limit of 0.333 μg/mL and could be reused for more than 13 cycles.

In a similar approach, the concentration of gliadin [73] in different foodstuffs was evaluated using QCM electrodes modified with 25 mM AuNPs. The chip was functionalized with glutaraldehyde followed by the reaction of the carbonyl groups on the chip with cysteamine. The AuNPs were linked to the bound cysteamine through the sulfhydryl groups and after a new addition of cysteamine, the anti-gliadin antibodies were attached to the surface of AuNPs using glutaraldehyde. The proposed chip modification chip increased the number of immobilized antibodies significantly in comparison with a non-modified QCM electrode, as confirmed by the increase observed in the frequency shift (48%) for the analysis of the same concentration of gliadin (2 ppm). The biosensor showed a detection limit of 8 ng/mL in 60% ethanol, which increased to 1 mg/L in food samples due to food matrix effects. The total analysis time was around 40 min.

**Figure 9:**
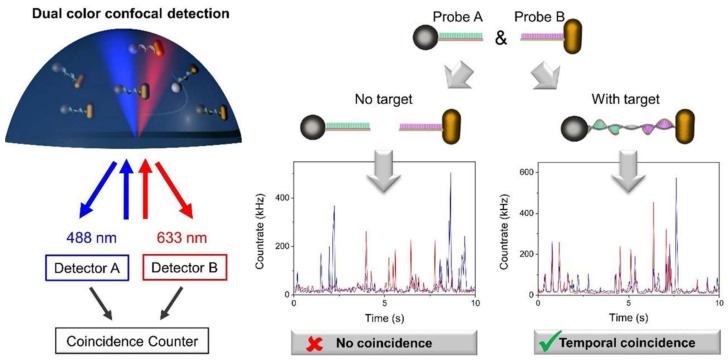
Principle of photon cross-correlation spectroscopy using AgNPs and AuNPs: the sample containing the probe metal NPs is illuminated by two lasers at different wavelengths (488 nm-blue and 633 nm-red). The resulting scattering signal is acquired to detect temporal coincidences between both signals. In the presence of the target DNA, nanoparticle probes A and B yielded temporal coincidence. Reprinted with permission from [123]. Copyright 2018 Wiley.

Vial et al. [123] introduced a novel technique for one-step detection of specific DNA sequences of sesame in homogeneous assay format. The sensing approach was based on detecting the light intensity scattered by individual metal Au and Ag aggregates at two different wavelengths. The authors named it as photon cross-correlation spectroscopy (Figure 9).

The sensing mechanism was based on the hybridization between the ssDNA target and the ssDNA probes anchored to the NP surfaces. In the presence of the target DNA, the AgNP spheres and AuNP rods became linked to each other, forming an aggregate that diffused simultaneously across the analysis volume, yielding temporal coincidences between the scattered signals. The level of correlation between both signals allowed quantification of the target DNA in the range of 5 pM to 1.5 nM, after 30 min incubation at 65 °C. The limit of detection was 1 pM and biosensor specificity was excellent, which allowed the detection of single nucleotide polymorphisms. The authors stated that this approach is fast, simple to operate and shows promising application prospects for point-of-care biosensors.

## 5. Conclusions

The prevalence and severity of food allergies is increasing steadily worldwide. Research in this field has focused on different aspects such as clinical diagnostics, evaluation of the effectiveness of cleaning practices in the food industry, or better definition of the threshold doses. From the analytical point of view, new methods are required for the detection of trace concentrations of allergens in complex food matrices as well as for hidden food allergens or new allergenic foods not commercially available yet. In this regard, biosensors can be of interest for both the food industry and the regulatory bodies responsible for the protection of the food-allergic consumers’ health. The application of nanomaterials to the development of biosensors for food analysis has led to improved sensitivity, selectivity, robustness and accuracy of conventional analytical methods. Moreover, nanobiosensors usually enable higher efficiency and sample throughput, as well as a better performance in the analysis of complex food samples.

However, in the last years, relatively few sensors based on NPs have been described for the analysis of food allergens. Therefore, this approach should be further explored applying different transduction principles. Hereof, the combination of new nanosized materials, with unique or novel properties, with new selective biorecognition elements, shows excellent prospects for the development of portable, miniaturized, real-time, easy-to-use, rapid and cost-effective sensors capable of screening multiple allergens if required. Other challenges in this field include the search of new ways to enhance the signal-to-noise ratio, signal amplification, enhancement of the transduction efficiency, sample preparation and biosensor validation. In any case, it is crucial to bridge the gap between laboratory research and the real world. The food industry may play an essential role in this regard, encouraging research in this field and adopting the resulting devices in routine food testing.

## Figures and Tables

**Table 1:** Ingredients listed in the Annex IIIa of Directive 2003/89 that must be labelled.

Commission Directive 2003/89	Commission Directive 2005/26	Regulation (EU) No 1169/2011
Cereals containing gluten (i.e., wheat, rye, barley, oats, spelt, kamut or their hybridized strains) and products thereof	Cereals containing gluten (i.e., wheat, rye, barley, oats, spelt, kamut or their hybridized strains) and products thereof, except: - Wheat-based glucose syrups including dextrose ^(1)^ - Wheat-based maltodextrins ^(1)^ - Glucose syrups based on barley - Cereals used for making distillates or ethyl alcohol of agricultural origin for spirit drinks and other alcoholic beverages	Cereals containing gluten, namely: wheat, rye, barley, oats, spelt, kamut or their hybridized strains and products thereof, except: - Wheat-based glucose syrups including dextrose ^(1)^ - Wheat-based maltodextrins ^(1)^ - Glucose syrups based on barley - Cereals used for making alcoholic distillates including ethyl alcohol of agricultural origin
Crustaceans and products thereof	Crustaceans and products thereof	Crustaceans and products thereof
Eggs and products thereof	Eggs and products thereof	Eggs and products thereof
Fish and products thereof	Fish and products thereof, except: - Fish gelatine used as carrier for vitamin or carotenoid preparations - Fish gelatine or Isinglass used as fining agent in beer and win	Fish and products thereof, except: - Fish gelatine used as carrier for vitamin or carotenoid preparations - Fish gelatine or Isinglass used as fining agent in beer and wine
Peanuts and products thereof	Peanuts and products thereof	Peanuts and products thereof
Soybeans and products thereof	Soybeans and products thereof, except: - Fully refined soybean oil and fat ^(1)^ - Natural mixed tocopherols (E306), natural d-α-tocopherol, natural d-α-tocopherol acetate, natural d-α-tocopherol succinate from soybean sources - Vegetable oils derived phytosterols and phytosterol esters from soybean sources - Plant stanol ester produced from vegetable oil sterols from soybean sources	Soybeans and products thereof, except: - Fully refined soybean oil and fat ^(1)^ - Natural mixed tocopherols (E306), natural d-α-tocopherol, natural d-α-tocopherol acetate, natural d-α-tocopherol succinate from soybean sources - Vegetable oils derived phytosterols and phytosterol esters from soybean sources - Plant stanol ester produced from vegetable oil sterols from soybean sources
Milk and products thereof (including lactose)	Milk and products thereof (including lactose), except: - Whey used for making distillates or ethyl alcohol of agricultural origin for spirit drinks and other alcoholic beverages - Lactitol	Milk and products thereof (including lactose), except: - Whey used for making alcoholic distillates including ethyl alcohol of agricultural origin - Lactitol
Nuts i.e., Almond (*Amygdalus communis* L.), Hazelnut (*Corylus avellana*), Walnut (*Juglans regia*), Cashew (*Anacardium occidentale*), Pecan nut (*Carya illinoiensis* (Wangenh.) K. Koch), Brazil nut (*Bertholletia excelsa*), Pistachio nut (*Pistacia vera*), Macadamia nut and Queensland nut (*Macadamia ternifolia*) and products thereof	Nuts, i.e., almonds (*Amygdalus communis* L.), hazelnuts (*Corylus avellana*), walnuts (*Juglans regia*), cashews (*Anacardium occidentale*), pecan nuts (*Carya illinoiesis* (Wangenh.) K. Koch), Brazil nuts (*Bertholletia excelsa*), pistachio nuts (*Pistacia vera*), macadamia nuts and Queensland nuts (*Macadamia ternifolia*) and products thereof, except: - Nuts used for making distillates or ethyl alcohol of agricultural origin for spirit drinks and other alcoholic beverages	Nuts, namely: almonds (*Amygdalus communis* L.), hazelnuts (*Corylus avellana*), walnuts (*Juglans regia*), cashews (*Anacardium occidentale*), pecan nuts (*Carya illinoinensis* (Wangenh.) K. Koch), Brazil nuts (*Bertholletia excelsa*), pistachio nuts (*Pistacia vera*), macadamia or Queensland nuts (*Macadamia ternifolia*) and products thereof, except for nuts used for making alcoholic distillates including ethyl alcohol of agricultural origin
Celery and products thereof	Celery and products thereof	Celery and products thereof
Mustard and products thereof	Mustard and products thereof	Mustard and products thereof
Sesame and seeds and products thereof	Sesame and seeds and products thereof	Sesame and seeds and products thereof
Sulfur dioxide and sulphites at concentrations of more than 10 mg/L or 10 mg/L expressed as SO_2_	Sulfur dioxide and sulphites at concentrations of more than 10 mg/L or 10 mg/L expressed as SO_2_	Sulfur dioxide and sulphites at concentrations of more than 10 mg/kg or 10 mg/L in terms of the total SO_2_ which are to be calculated for products as proposed ready for consumption or as reconstituted according to the instructions of the manufacturers
	Lupin and products thereof	Lupin and products thereof
	Molluscs and products thereof	Molluscs and products thereof

^(1)^ And products thereof, in so far as the process that they have undergone is not likely to increase the level of allergenicity assessed by the EFSA for the relevant product from which they originated.

**Table 2:** Major food allergens from plant sources [8,25,26].

Protein Family	Representative Allergenic Proteins
**Cupin superfamily**	
- Legumins (11S seed storage proteins)	Ara h 3 and Ara h 4 (peanut), glycinin subunits (soybean), Cor a 9 (hazelnut), AMP (almond)
- Vicilins (7S seed storage proteins)	Ara h 1 (peanut), α-subunit of β-conglycinin (soybean), Jug r 2 (English walnut), Len c 1 (lentil), Ana o 1 (cashew), Ses i 3 (sesame)
**Prolamin superfamily**	
- α-amylase/protease inhibitors	Hor v 15 (barley), Sec c 1 (rye), RAPs (rice allergenic proteins)
- Non-specific lipid transfer proteins	Pru p 3 (peach), Mal d 3 (apple), Pru ar 3 (apricot), Cor a 8 (hazelnut), Aspa o 1 (asparagus), Lac s 1 (lettuce)
- Storage proteins of cereals	Tri a 19 (wheat), Sec c 20 (rye), Hor v 21 (barley)
- 2S albumins	Sin a 1 (yellow mustard), Ber e 1 (Brazil nut), Jug r 1 (English walnut), Ses i 2 (sesame), Ara h 2, Ara h 6, Ara h 7 (peanut)
**Papain superfamily of cysteine proteases**	
- Papain-like cysteine proteases	Act c 1 (kiwi), papain (papaya), bromelain (pineapple), P34/Gly m Bd 30 K (soybean)
**Bet v 1-related protein**	Api g 2 (celery), Dau c 1 (carrot), Pyr c 1 (pear)
**Profilin**	Ara h 5 (peanut), Lyc e 1 (tomato), Mus a 1 (banana), Mal d 4 (apple), Pru av 4 (sweet cherry), Apig 4 (celery), Cor a 4 (hazelnut), Gly m 3 (soybean), Ana c 1 (pineapple)
**Kunitz-type protease inhibitors**	20 kDa Kunitz soybean trypsin inhibitor, Sola t 2, Sola t 3, Sola t 4 (potato)
**Lectins**	31 kDa peanut agglutinin
**Patatin-like proteins**	Sola t 1 (potato)
**Phenylcoumaran benzylic ether reductases**	Pyr c 6 (pear)
**Oleosins**	Peanut oleosin
**β-fructofuranosidases**	Lyc e 2 (tomato)
**Subtilisin-like serine proteases**	Cuc m 1/cucumisin (melon)
**FAD-containing oxidases**	Api g 5 (celery)

**Table 3:** Major food allergens from animal sources [25,26].

Food	Protein Family	Examples of Allergens
Cow’s milk	Lipocalins α/β-caseins Glycoside hydrolase family 22 Transferrins	β-lactoglobulin (Bos d 5) Casein (Bos d 8) α-lactalbumin (Bos d 4) Lactoferrin
Egg	Kazal-type serine protease inhibitors Serpins Transferrins Glycoside hydrolase family 22	Ovomucoid (Gal d 1) Ovalbumin (Gal d 2) Ovotransferrin (Gal d 3) Lysozyme (Gal d 4)
Fish	Calcium-binding EF-hand proteins	Parvalbumins: Gad c 1 (cod) Gad m 1 (cod) Sal s 1 (salmon) Cyp c 1 (carp)
Seafood	Tropomyosins ATP: guanido phosphotransferases	Pen i 1 (Indian shrimp), Par f 1 (Taiwanese shrimp), Cha f 1 (common crab), Pan s 1 (spiny lobster), Hom a 1 (American lobster), Cra g 1 and Cra g 2 (Pacific oyster), Tod p 1 (squid), Per v 1 (tropical green mussel) Arginine kinases: Par f 1 (Parapenaeus fissurus shrimp) Pen m 2 (Penaeus monodon shrimp)

**Table 4:** Electrochemical and piezoelectric biosensors based on NPs for the detection of food allergens.

Analyte	Sample	Nanomaterial	Transduction	Assay	Receptor	LOD	Ref.
Peanut Ara h 1	Peanuts	Spongy gold film/CS-MWCNT/GCE	Chronoamperometry	Direct	Stem-loop DNA	4.1 × 10^−8^ nmol/L	[69]
Peanut Ara h 1	Cookies and chocolate	SPCE-AuNPs	Voltammetry	Sandwich	Monoclonal anti-Ara h 1 2C12 antibody	3.8 ng/mL	[63]
Peanut Ara h 6	Cookies and chocolate	SPCE-AuNPs	Voltammetry	Sandwich	Monoclonal anti-Ara h 6 IgG (3B8 B5)	0.27 ng/mL	[63]
Peanut Ara h 2 Antibody	Serum	GSH-AuNP-coated electrode	f-EIS and nf-EIS	Direct	Ara h 2 antigen	5 pg/mL (nf-EIS)	[67]
Peanut protein Ara h 1	-	3 nm Au-coated pores of commercial nanoporous polycarbonate membranes	nf-EIS	Direct	Polyclonal antibody peanut protein	-	[72]
Casein	Cheese	AuNPs/P-l-Arg/MWCNTs/GCE	DPV	Indirect	Monoclonal anti-casein antibody	5 × 10^−8^ g/mL	[62]
Gliadin	Rice, corn, barley, rye, buckwheat, oats, mile, chestnut, chickpeas, quinoa, etc.	SPCE-AuNPs	DPV	Indirect	Monoclonal anti-gliadin antibody	8 ng/mL	[70]
Gliadin	Wheat, barley, oat, rice, foxtail millet, corn, buckwheat, soybean, pancake mix, custard mix, baby rice, etc.	5 nm AuNPs	QCM	Direct	Polyclonal chicken anti-gliadin antibodies	8 ng/mL	[73]
Shrimp Pen a1 and fish parvalbumin	Crucian carp and brown shrimp	Fe_3_O_4_@SiO_2_@FITC	f-EIS	Direct	RBL-2H3 mast cells	0.03 μg/mL (shrimp Pen a1) and 0.16 ng/mL (fish PV)	[71]
Shrimp allergen	Spiked water samples	10 nm AuNPs	QCM	Direct	Polyclonal anti-shrimp antibodies	0.333 µg/mL	[74]
Shrimp Pen a1	-	AuNPs-L-Cys-modified gold electrode	f-EIS	Direct	RBL-2H3 mast cells	0.15 μg/mL	[75]

Abbreviations: CS, chitosan; DPV, differential pulse voltammetry; f-EIS, faradaic electrochemical impedance spectroscopy; nf-EIS: non-faradaic electrochemical impedance spectroscopy; FITC, fluorescein isothiocyanate; GCE, glassy carbon electrode; GHS, glutathione reduced; MWCNT, multiwalled carbon nanotubes nanocomposite; SPCE, screen-printed carbon electrode; QCM, quartz crystal microbalance.

**Table 5:** Colorimetric nanoparticle-based assays for the detection of food allergens.

Analyte	Sample	Nanomaterial	Transduction	Assay	Receptor	LOD	Ref.
Ara h 1	Peanut samples, cookies, chocolates, hazelnuts, legumes	Colloidal AuNP-antibody conjugate	Absorbance (coloured red response, naked eye)	Sandwich immunoassay	2 mAbs anti-Ara h 1	10 ng/mL	[86]
β-lactoglobulin	Crude milk extract, cookies, chocolates, corn bar, rusk	Colloidal AuNP-antibody conjugate	Absorbance (coloured red response, naked eye)	Sandwich immunoassay	2 mAbs anti-β-lactoglobulin	0.2 ng/mL	[87]
Parvalbumin	*Linnaeus*, fish balls, fish tofu, beef, pork and chicken	13 nm AuNPs and AuNPs−antibody conjugate	Absorbance (brightness for semi quantitative analysis, VDL)	Direct competitive immunoassay (microarray)	Anti-parvalbumin antibody	70 ng/mL (VDL, qualitative) 40 ng/mL (semi quantitative LOD)	[88]
Gliadin Casein β-Lactoglobulin Ovalbumin	Baby foods Juices Beers	5 nm AuNPs-antibody conjugate	Absorbance (precipitate OD-Ag mediated)	Multiplex competitive immunoassay	mAbs for gliadin, casein and ovalbumin; pAb for β-lactoglobulin	0.04, 0.40, 0.08 and 0.16 mg/L (in terms of EC_50_)	[91]
Ara h 3/4	Biscuits and cereals	2.8 µm Magnetic microparticles- dendrimer-antibody conjugate	Absorbance (650 nm)	Indirect immunoassay	mAb anti-Ara h 3/4	0.2 mg/kg	[92]
Gliadin	-	20 nm AuNPs-goat anti-rabbit IgG-HRP conjugate	Absorbance (450 nm)	Indirect immunoassay	Rabbit anti-gliadin pAb	180 pg/mL	[93]

Abbreviations: AuNP, gold nanoparticle, mAb, monoclonal antibody; pAb, polyclonal antibody; OD, optical density; EC_50_, half maximal effective concentration; REA, Resonance-enhanced absorption; IgG, Immunoglobulin G, OVA, ovalbumin; OVO, ovomucoid; HRP, horseradish peroxidase; VDL, visual detection limit.

**Table 6:** Fluorescence, surface-enhanced Raman scattering (SERS) and other optical-based bioassays for the detection of food allergens using NPs.

Analyte	Sample	Nanomaterial	Transduction	Assay	Receptor	LOD	Ref.
Bovine α-Lactoglobulin (α-La)	Milk	QDs (CdSe/ZnS) coated with anti-α-LA	Fluorescence (FLISA)	Competitive immunoassay	mAb anti-α-La	0.1 ng/mL (DR: 0.1–1000 ng/mL, EC_50_: 0.03 mg/mL)	[101]
Ara h 1	Peanut	Bypyramid-shaped gold nanocrystals (BPGNs) bound to thiolated DNA molecular beacon (MB) Cy3-labeled	SERS	Displacement DNA assay	Thiolated DNA MB Cy3-labeled	3.3 10^−15^ M (S/N = 3) (DR: 10^−14^–10^−8^ M)	[121]
Ovalbumin (OVA), ovomucoid (OVO)	Whole egg powder, hydrolysed egg, pasta, dessert, cheese and tomato	16 nm AuNPs−antibody conjugate	REA	Competitive inhibition immunoassay (OVO, OVA) and sandwich immunoassay (OVO)	pAb anti-OVA and pAb anti-OVO	Semi quantitative 1 ng/mL (DR: 1 ng/mL–1 mg/mL)	[84]
Ara h 1	Candy bar	AuNPs decorated with anti-Ara h 1	Fibre optic-SPR (FO-SPR)	Sandwich immunoassay	Biotinylated Ara h 1 aptamer	75 nM (DR: 0–634 nM)	[114]
Ara h 1	Candy bar	MNPs coated with anti-Ara h 1	Fibre optic-SPR (FO-SPR)	Sandwich immunoassay	Primary and secondary pAbs anti-Ara h 1	0.1 μg/mL (DR: 0–40 μg/mL Regenerated 35 cycles)	[65]
Anti-allergen IgG antibodies for Ara h 1 and for other allergens of non-food origin	Buffer	AuNPs deposited in a flow cell	NSMA (Localized plasmon array biosensor)	Direct immuno-kinetic assay	Protein Ara h 1 + anti-IgG	2 nM	[124]
Tropomyosin (shrimp allergen)	Buffer	Graphene oxide NPs	Fluorescence (GO/ssDNA-oliGreen quenching system)	Displacement assay	Tropomyosin DNA-aptamer	4.2 nM (DR: 0.5–50 μg/mL, no CR with BSA, SA, lysozyme, thrombin, myosin, etc.)	[106]
Ara h 1 peanut allergen	Biscuit	Graphene oxide NPs and QDs	Fluorescence (QDs-aptamer/GO quenching system)	Displacement assay (microfluidic platform)	Ara h 1-DNA aptamer	56 ng/mL (RD: 200–2000 ng/mL, no CR with Ara h 2 and Ara h 3)	[107]
Allergens of peanuts, egg	Buffer	40 nm AuNPs coated with specific anti-allergen	REA (metal nanocluster resonance)	Sandwich immunoassay (microarray)	Primary and secondary pAbs anti-allergen	500 μg/mL (peanut), 100 μg/mL (egg)	[125]
Bovine β-Lactoglobulin (β-La)	UHT-milk, whey, wheat, chocolate, cookies	CdTe QDs	Fluorescence (QDs/H_2_O_2_-SACat quenching system)	Sandwich immunoassay	mAb anti-β-La + biotinylated pAb anti-β-La	0.49 ng/mL (DR: 0.49–62.5; 125–4000 ng/mL, no CR with egg, peanut, soy wheat proteins, weak CR with casein)	[102]
Casein	Milk	GMNPs coated with anti-casein mAb + CdTe QDs coated with anti-casein pAb	Fluorescence (Intensity @ 620 nm)	Sandwich two-bead immunoassay	mAb anti-casein + pAb anti-casein	EC_50_ 36.59 ng/mL (DR: 4.617–289.9 ng/mL, no CR with α-La, β-La, BSA and OVA)	[126]
Lysozyme	Egg	Dendritic AgNPs	SERS	Direct assay (label-free)	Thiolated Lysozyme DNA-aptamer	0.5 μg/mL(water) 5 μg/mL (stainless steel food-handling surface) (DR: 0–6 μg/mL)	[122]
Casein	Milk	Silica NPs covered with a gold layer in which anti-casein is immobilized	LSPR	Direct immunoassay (label free)	pAb anti-casein	10 ng/mL (DR: 0.1–10 μg/mL, no CR with α-La and β-La)	[116]

Abbreviations: FLISA, fluorescence-linked immunosorbent assay, SERS, Surface-enhanced Raman spectroscopy, REA, Resonance-enhanced absorption; SPR, Surface-plasmon Resonance, IRIS, Interferometric Reflectance Imaging Sensor, DLS, dynamic light scattering, Photo-CCS, photon cross-correlation spectroscopy, NSMA, nanoparticle scattering multiplexing assay, LSPR, localized surface plasmon resonance, AuNPs, gold nanoparticles, AgNPs, silver nanoparticles, MNPs, magnetic nanoparticles, MB, molecular beacon; pAb, polyclonal antibody; mAb, monoclonal antibody OD, optical density; EC_50_, half maximal effective concentration; IgG, Immunoglobulin G, OVA, ovalbumin; OVO, ovomucoid; HRP, horseradish peroxidase; DR, dynamic range, CR, cross reactivity, GO, graphene oxide, SA-Cat, catalase streptavidin conjugate, GMNPs, gold magnetic nanoparticles.
